# Innocent Laryngeal Growths.—I

**Published:** 1908-12-19

**Authors:** 


### Laryngology and Rhinology.

### INNOCENT LARYNGEAL GROWTHS.—I.

Our knowledge of the nature and ætiology of the commoner forms of innocent tumours of the larynx is in certain respects somewhat incomplete, and it follows that their classification is not easily made on exact scientific principles. With regard to some of these growths, irritation appears to be the primary cause, especially of such of them as occur at the centre of the vocal cord, and all gradations are found between simple inflammatory thickening and definite tumour formation. It is probable, also, that some of them are produced by the irritative action of micro-organisms, and it is well known that both tuberculosis and syphilis may be the cause of a growth clinically, and even microscopically, indistinguishable from a papilloma. On the other hand, these neoplasms are not very uncommon in early childhood, and are found in parts of the larynx not especially subject to irritation. A few of them, especially those occurring at the anterior commissure, may develop from the remnants of embryonic tissue.

### Singer's Nodule.

Singer's nodule, which is the most frequently encountered of these growths on the vocal cord, lies on the borderland between true neoplastic formation and simple inflammatory thickening. The first stage in its formation is a proliferation of the epithelium, followed by an increase of the subepithelial tissue in which appear at a later stage spaces containing fluid; these spaces may be the beginning of the formation of a cyst or of myxomatous degeneration. As usually seen, however, these little growths must be classed rather with chronic inflammation and pachydermatous thickening. They occur especially in women and, notwithstanding their name, are not often found in trained singers, but far more frequently in those who use the speaking voice daily for long periods at a time. They are consequently very common in school-teachers.

The principal factor in the causation of these nodules is faulty production of the voice, especially rigidity and over action of the muscles of the throat and insufficient use of the nasal resonance; nasal disease, and especially nasal obstruction, therefore becomes of importance by making proper voice-production impossible. The use of ill-ventilated and dusty rooms also has a bad effect and in this connection the chalk from the blackboard may be mentioned as very deleterious to the throats of teachers; a damp sponge should always be employed instead of the usual dry cloth. These growths invariably occur at the centre of the true vocal cord; that is, at the junction of the anterior and middle thirds of the entire glottis, which includes the vocal process of the arytenoid. This spot is the "seat of election" for innocent tumours of the cord. It is sometimes considered to be a nodal point, but it is not so; on the contrary, it is the point of maximum vibration. The two cords should never come in contact on phonation, but should lie parallel and close to each other; if, however, they touch by reason of faulty action or of swelling, it is at this point that the greatest irritation will occur. In many cases of laryngeal catarrh a little globule of mucus forms at this spot during phonation; this may even be mistaken for a tiny nodule, but that it disappears on coughing.

The ætiology of singer's nodule has been considered rather fully because it is identical with that of the other common innocent growths of the vocal cord, and because the understanding of their mode of origin is essential to the rational treatment of all these growths, both before removal and to prevent recurrence. It has been stated already that there is no distinct line of demarcation between these neoplasms and inflammatory thickening. Similar nodules are, however, occasionally seen in children and even young infants, and these tend to disappear at puberty. Papillomata, also, as is well known, occur not uncommonly in children, and may grow on any part of the larynx, though when a single papilloma occurs on the vocal cord it is nearly always found at the seat of election.

### Fibromata and Papillomata.

A little firm globular growth is not uncommon at the seat of election on the vocal cord, and is in all probability simply a further development of the singer's nodule. In colour it may be white, pink, red, or brown from extravasation of blood; it is pedunculated and its size varies up to that of a pea. Fibromata of quite another type have been very rarely recorded; they may grow from any part of the larynx, are irregular in shape, and may reach a relatively enormous size, filling the larynx and causing asphyxia. So-called myxomata are described; they are merely instances of myxomatous degeneration of fibromata and are similar in appearance, but softer and more translucent, and resemble a small nasal polypus in appearance.

Papillomata are the commonest of all innocent growths of the larynx, and occur at all ages. They are found in children and young infants, sometimes even appearing to be congenital; and they are also seen in old men, when they very naturally excite the suspicion of malignancy. A single papilloma usually occupies the centre of the vocal cord, and they are rarely seen at the posterior part of the glottis; but papillomata may occur on any part of the larynx and are often multiple, more especially in children. They are soft in texture, red in colour, papillary in shape, and are pedunculated; they do not bleed spontaneously. Multiple papillomata show a decided tendency to recur after removal, or rather they continue to appear though not necessarily on the same site. Like warts on other parts of the body, they sometimes vanish spontaneously with great rapidity, and when occurring in childhood are said to show a tendency to disappear at puberty.

Other innocent growths are rare; adenomata and lipomata have been recorded; cysts occur chiefly on the epiglottis and ary-epiglottic folds, but some of the little growths at the seat of election are cystic. Angeiomata are rare, but important by reason of the liability to hæmorrhage; they take the form of a flat purple patch or a definite raised tumour and occur on the vocal cords or on any part of the larynx.

(*To be continued.*)

